# DT2008: A Promising New Genetic Resource for Improved Drought Tolerance in Soybean When Solely Dependent on Symbiotic N_2_ Fixation

**DOI:** 10.1155/2015/687213

**Published:** 2015-01-05

**Authors:** Saad Sulieman, Chien Van Ha, Maryam Nasr Esfahani, Yasuko Watanabe, Rie Nishiyama, Chung Thi Bao Pham, Dong Van Nguyen, Lam-Son Phan Tran

**Affiliations:** ^1^Signaling Pathway Research Unit, RIKEN Center for Sustainable Resource Science (CSRS), 1-7-22 Suehiro-cho, Tsurumi, Yokohama 230-0045, Japan; ^2^Department of Agronomy, Faculty of Agriculture, University of Khartoum, 13314 Shambat, Khartoum North, Sudan; ^3^National Key Laboratory of Plant Cell Biotechnology, Agricultural Genetics Institute, Vietnamese Academy of Agricultural Science, Hanoi 100000, Vietnam; ^4^Department of Biology, Lorestan University, Khorramabad 68151-44316, Iran; ^5^Department of Mutation and Heterosis Breeding, Agricultural Genetics Institute, Vietnamese Academy of Agricultural Science, Hanoi 100000, Vietnam

## Abstract

Water deficit is one of the major constraints for soybean production in Vietnam. The soybean breeding research efforts conducted at the Agriculture Genetics Institute (AGI) of Vietnam resulted in the development of promising soybean genotypes, suitable for the drought-stressed areas in Vietnam and other countries. Such a variety, namely, DT2008, was recommended by AGI and widely used throughout the country. The aim of this work was to assess the growth of shoots, roots, and nodules of DT2008 versus Williams 82 (W82) in response to drought and subsequent rehydration in symbiotic association as a means to provide genetic resources for genomic research. Better shoot, root, and nodule growth and development were observed in the cultivar DT2008 under sufficient, water deficit, and recovery conditions. Our results represent a good foundation for further comparison of DT2008 and W82 at molecular levels using high throughput omic technologies, which will provide huge amounts of data, enabling us to understand the genetic network involved in regulation of soybean responses to water deficit and increasing the chances of developing drought-tolerant cultivars.

## 1. Introduction

Soybean (*Glycine max *(L.) Merr.) has been classified among the most important commercial oilseed crops worldwide [1]. It can substantially provide oils, micronutrients, minerals, and vegetable proteins suitable for livestock feed and human consumption. In addition, soybean has supplied materials for industrial uses, such as biodiesel, plastics, lubricants, and hydraulic fluids. Currently, world production of soybean is greater than any other oilseed crop. Globally, it accounts for approximately 68% of global crop legume production and 57% of world oilseed production [2]. Collectively, soybean production occupies around 6% of the world's available land [3].

As a leguminous plant, soybean has a superior potential capability to fix atmospheric N_2_ in association with highly specialized soil bacteria. Under most conditions, soybean meets 58–68% of its nitrogen (N) demand through symbiotic association, but it can fulfill up to 100% with the aid of this vital process [4–6]. Moreover, a large portion of the fixed N can be readily accessible for the subsequent crops in the rotation systems or the natural ecosystems. Therefore, the soybean-rhizobia relationship represents a vital option to sustain agricultural development due to its superior N_2_ fixation, enabling us to reduce the dependence on N fertilizers and thus avoiding the overexploitation of natural resources. Optimizing this association can upgrade soybean production and enhance soil fertility, whilst reducing the production costs and environmental impacts associated with N-chemical fertilizers [5]. Nevertheless, nodulating soybean plants growth and production are highly sensitive to adverse environmental conditions, particularly water scarcity in soils [7, 8].

In Vietnam, soybean occupies an important front position in the structure of agricultural crops throughout the country [9]. Recently, Vietnam's soybean production continues to fall well below the demand for food, feed, and vegetable oil industry. According to the 2012's statistical data, Vietnam imported 1.29 million metric tonnes of soybeans which represents a 26% increase over the previous year [10]. Due to high prices in the global market, soybean importation value had reached $776 million in 2012 (41% increase over the past year). Currently, the Vietnamese Government's Master Plan for Oilseeds has further development priorities for the sector with an objective of 350000 ha of soybean-cultivated land and a production of 700000 metric tonnes by 2020 (http://www.thecropsite.com/reports/?id=3701&country=VN). However, drought has a tremendous effect on soybean growth and development, thus negatively affecting the projected expansion of crop production [11]. In recent years, drought has occurred more and more commonly as a result of global warming and climate change [12]. Therefore, selective breeding for high drought-tolerant soybean cultivars and investigating the mechanisms to improve the drought tolerance of soybean have become top priority for many scientific researchers. On this basis, the soybean breeders at the Agriculture Genetics Institute (AGI) of Vietnam have initiated a long-term soybean breeding program to construct and release various drought-tolerant soybean cultivars through conventional breeding and radiation-induced mutagenesis. One of the newly developed prospective cultivars, the DT2008, revealed enhanced drought tolerance capability and yield stability (~2–4 metric tonnes per ha) under various field growing conditions [13]. Thus, we have started a joint project to fully characterize this cultivar under drought and various N regimes. Under nonnodulation conditions, we have recently documented that DT2008 has higher drought tolerance ability against the soybean reference cultivar Williams 82 (W82) [14].

In this report, we have extended our previous approach by comparing the drought-tolerant cultivar DT2008 and W82 based on their potential symbiotic association under drought and rehydration treatments. Results of this study demonstrated that DT2008 has a better drought tolerance and higher recovery level than W82.

## 2. Materials and Methods

### 2.1. Biological Materials

The soybean variety DT2008 was basically created by multiple hybridizations of local cultivars and subsequent irradiation with gamma rays Co^60^ − 18 Gy + F4 (DT2001/IS10) [15]. It has a wide adaptability to various harsh conditions and is suitably cultivated in 3 crops per year with a growth duration ranging from 110 to 120 days and relatively higher yield potentiality (2.5–4.0 tonnes/ha). In addition to its superior drought and thermotolerance, DT2008 has comparatively higher level of resistance against three kinds of diseases, namely, rust, downy mildew, and bacterial pustule [15]. Thus, in this study, we have intended to examine this promising drought-tolerant variety versus the widely used soybean reference cultivar W82 that was used to produce the reference genome sequence of soybean [16]. Accordingly, these materials would provide an efficient platform for omic analyses to identify new single nucleotide polymorphisms (SNPs) and promising candidate genes for genetic engineering. For testing the potential symbiotic capability under drought and rehydration conditions, both cultivars were inoculated with the microsymbiont* Bradyrhizobium japonicum *strain USDA110. Owing to its superior symbiotic N_2_ fixation activity and full determination of its genome sequence,* B. japonicum *USDA110 has been widely used for the purpose of physiology, molecular genetics, and ecological studies [17].

### 2.2. General Plant and Bacterial Growth Conditions

Seeds of DT2008 and W82 were separately germinated in 6-litre pots containing autoclaved vermiculite as rooting substrate in a controlled greenhouse conditions (continuous 30°C temperature, 60% relative humidity, 12/12 h photoperiod, and 150 *μ*mol m^−2^ s^−1^ photon flux density). Seeds were inoculated with* B. japonicum* USDA110 grown in yeast mannitol broth (YMB) (mannitol 2 g L^−1^; yeast extract 0.4 g L^−1^; K_2_HPO_4_ 0.5 g L^−1^; MgSO_4_·7H_2_O 0.2  g L^−1^; NaCl 0.1 g L^−1^; pH 6.8) for 48 h at 28°C. Cultures were diluted with water and added at a rate of ~10^8^ cells mL^−1^ after the seeds were sown in the vermiculite. Plants were watered to field capacity three times a week with full-strength Herridge's nutrient solution [18] until the stress treatments were imposed. The basal nutrient solution contained 0.25 mM CaCl_2_; 0.25 mM KCl; 0.5 mM MgSO_4_·7H_2_O; 0.13 mM KH_2_PO_4_; 0.13 mM K_2_HPO_4_; 23.5 *μ*M Fe (III)-EDTA; 71.5 × 10^−2 ^mg L^−1^H_3_BO_3_; 45.3 × 10^−2 ^mg L^−1^MnCl_2_·4H_2_O; 2.8 × 10^−2 ^mg L^−1^ZnCl_2_; 1.3 × 10^−2 ^mg L^−1^CuCl_2_·2H_2_O; and 0.6 × 10^−2 ^mg L^−1^ NaMoO_4_·2H_2_O.

### 2.3. Drought and Recovery Treatments

Drought was imposed on 21-day-old plants by withholding water. The plants were randomly separated into two main sets (control and drought) containing four biological replicates each. Control (well-watered) plants were watered every day, whereas drought was imposed by withholding water for either 4 or 7 days (4 D or 7 D). Recuperation was carried out by rewatering the stressed plants for 3 days (7 D + 3 W). Both water-stressed (WS) and well-watered (WW) plants were harvested at set time points: 4, 7, and 10 D after the onset of drought-rehydration treatments. At each time point, soil volumetric moisture contents (VMC) were monitored using a HydroSense soil moisture probe (Campbell Scientific, Inc.). Measurements of various growth and nodulation parameters were performed at the end of the stress and rehydration periods. At each harvest, plants were fractioned into shoots, roots, and nodules. Shoot and root tissues were dried at 65°C for a minimum of 48 h and weighed for dry matter (DM) determination.

### 2.4. Statistical Analysis

Means and standard errors (SEs) were used to plot figures and evaluate treatment responses. All statistical analyses were performed using statistical tools imbedded in Microsoft Excel 2010. The significance of difference between means was determined by Student's* t*-test where the values of *P* < 0.05 were considered significant and those of *P* < 0.01 and *P* < 0.001 were highly significant.

## 3. Results and Discussion

Drought is a recurring problem limiting nodulation and N_2_ fixation in crop production particularly in tropical and semiarid tropical areas [19, 20]. Under the present scenarios of climate change, drought is more likely to occur, leading to ultimate growth and productivity reductions for most important economic crops, including soybean. Although access to irrigation can be used partially to alleviate drought impact, the usage of soybean drought-tolerant cultivars remains the most practically promising strategy to adopt. To cope with water deficit, drought-tolerant cultivars have developed a number of strategies that are genetically encoded [1, 9]. Thus, it is important to elucidate these striking adaptive mechanisms developed in such tolerant cultivars in order to improve the agronomic performance of soybean and other plant species by genetic engineering [21, 22]. Indeed, many physiological and biochemical responses to drought are shared amongst various tested plant species [23].

Our adopted strategy in the improvement of drought-tolerant cultivars is based on establishing an integrated approach involving conventional breeding and radiation-induced mutagenesis program and subsequently analyzing the internal adaptive mechanisms, which underlie plant responses to drought, through molecular biology techniques. A long-term, multidisciplinary research program was started to produce high-yielding adaptive cultivars for limited water conditions which exploited the drought-tolerant traits of DT2008. This biological resource was basically produced by multiple hybridizations of local cultivars and irradiation exposure [14]. A question was then raised whether DT2008 is able to maintain N_2_ fixation at high level during drought, thus contributing to its improved productivity. To provide an answer to this question, in this study, we initially carried out a comprehensive comparative analysis between DT2008 and the reference cultivar W82, and whole genome was sequenced [16], thereby enabling us to identify potentially important mutations or SNPs responsible for enhanced N_2_ fixation under drought in future molecular studies.

### 3.1. Plant Growth and Biomass Production are Less Negatively Affected in the Drought-Tolerant Cultivar DT2008

The alteration in biomass allocation is a principle strategy for coping with progressive soil-drying conditions [14]. Several groups have reported that DM partitioning is very important in the determination of soybean productivity [19, 24]. Total biomass has been used as a selection criterion for assessing drought tolerance in soybean. Understanding assimilation and allocation processes affected by water deficit is a fundamental prerequisite step in identifying and improving soybean tolerance to drought [11].

**Figure 1:**
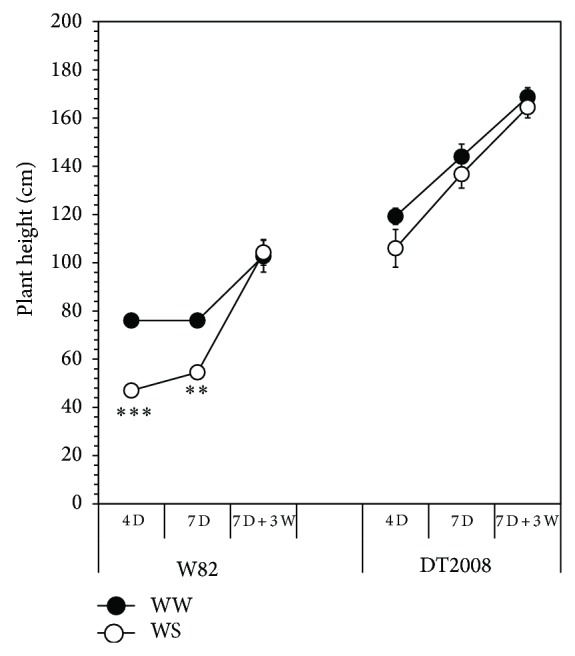
Comparison of plant height of the nodulated W82 and DT2008 plants after growth for 21 days in vermiculite soil and exposure to drought stress and recovery treatments. Well-watered (WW) plants were irrigated every day, whereas drought stress (WS) was imposed by withholding water for either four (4 D) or seven (7 D) days. Recovery treatment was experienced by withholding water for seven days followed by a subsequent rewatering for three days (7 D + 3 W). Error bars represent standard errors (*n* = 4 plants/genotype). Asterisks indicate significant differences as determined by Student's* t*-test ( ^**^ *P* < 0.01;  ^***^ *P* < 0.001).

In this study, soybean genotypes tested showed differential responses for growth traits examined. For example, the DT2008 plants exhibited more stable shoot growth in terms of shoot length (Figure 1), shoot fresh weight (FW), and dry weight (DW) (Table 1), when compared with W82, suggesting that DT2008 possesses a better shoot growth rate than W82 under the examined water deficit regimes (4 D, 7 D). In contrary, W82 exhibited a higher degree of susceptibility upon subjection to the equivalent drought treatments as indicated by significant decreases in the shoot length (Figure 1), shoot FW, and DW (Table 1).

**Figure 2:**
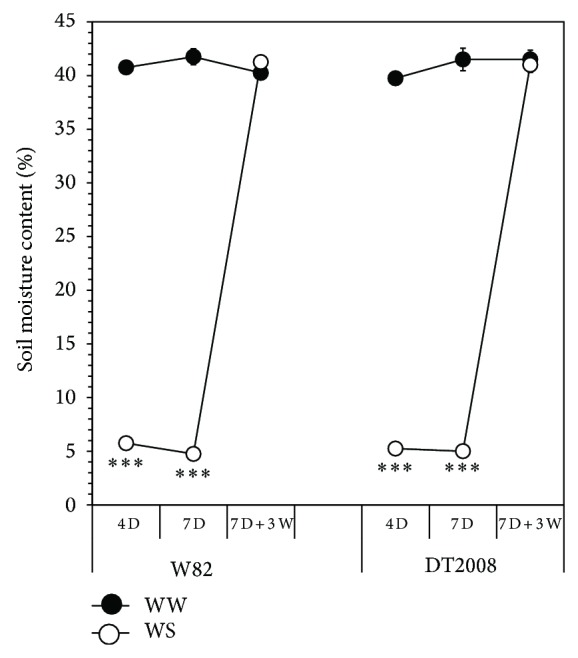
Monitoring of volumetric soil moisture contents during the drought stress and recovery treatments. Nodulated W82 and DT2008 plants were grown for 21 days in vermiculite soil and exposed to water deficit and rewatering treatments. Well-watered (WW) plants were irrigated every day, whereas drought stress (WS) was imposed by withholding water for either four (4 D) or seven (7 D) days. Recovery treatment was experienced by withholding water for seven days followed by a subsequent rewatering for three days (7 D  + 3 W). Error bars represent standard errors. Asterisks indicate significant differences as determined by Student's* t*-test ( ^***^ *P* < 0.001).

Drought tolerance mechanisms in leguminous plants are closely related to the root traits of the cultivated genotypes [23, 25]. In comparison with W82, DT2008 was more tolerant to water deficit as judged by its higher root fresh and DM biomass accumulations (Table 1). Understandably, maintenance of DT2008 root growth under progressive decline in soil water content would enhance drought tolerance due to an increased capacity of water uptake. Importantly, differences between DT2008 and W82 were observed under conditions that ensured similar amounts of soil water as indicated by VMC measurements (Figure 2). These results further support that DT2008 is more strongly tolerant to drought than W82 as indicated recently under nonnodulation conditions [14]. Collectively, our results suggest that the enhanced root systems of DT2008 may significantly contribute to its improved drought tolerance in comparison with W82.

### 3.2. Drought-Induced Changes in Nodulation Patterns

**Figure 3:**
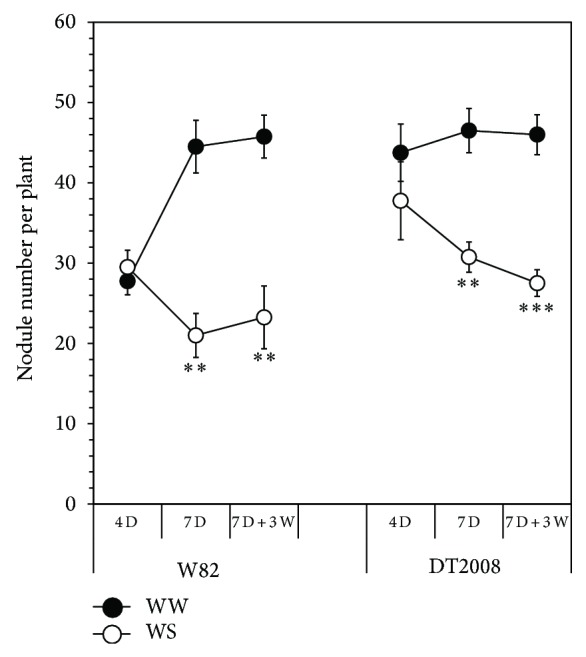
Comparison of nodule number per plant of the nodulated W82 and DT2008 plants after growth for 21 days in vermiculite soil and exposure to drought stress and recovery treatments. Well-watered (WW) plants were irrigated every day, whereas drought stress (WS) was imposed by withholding water for either four (4 D) or seven (7 D) days. Recovery treatment was experienced by withholding water for seven days followed by a subsequent rewatering for three days (7 D + 3 W). Error bars represent standard errors (*n* = 4 plants/genotype). Asterisks indicate significant differences as determined by Student's* t*-test ( ^**^ *P* < 0.01;  ^***^ *P* < 0.001).

Most leguminous plants, including soybean, have particular features in response to water deficit, such as reduced rates of nodulation and nitrogenase activity [26]. The acute sensitivity of nodulation to water deficit has been considered a major limiting factor towards improving soybean productivity. Despite several attempts and considerable research effort during the last decades, the molecular mechanism(s) underlining this sensitivity remains largely unidentified [27]. In this work, the effect of drought on the N_2_ fixation was evaluated based on nodule growth and development, specifically, the number of nodules per plant (Figure 3) and total nodule FW (Table 1), which frequently correlate well with shoot DM, providing an acceptable basis of N_2_-fixing efficiency [28].

In comparison with W82, DT2008 established relatively higher number of nodules per plant (Figure 3) and accumulated more nodule FW (Table 1) under sufficient water supply which might contribute to the higher growth rate of DT2008 versus that of W82 (Table 1). Upon exposure to water deficit (4 D and 7 D), nodulation pattern, in terms of nodule number, was found to be different between DT2008 and W82. The total nodule number per plant significantly reduced in both cultivars after 7 D of water stress; however, the nodule number in DT2008 was still significantly higher than that in W82 at 7 D (*P* < 0.05 as measured by Student's* t*-test) (Figure 3). In case of nodule FW, although the 4 D water stress regime resulted in a significant reduction of nodule FW in both W82 and DT2008, the nodule FW in the drought-tolerant DT2008 was still slightly higher than that of the sensitive W82 cultivar (Table 1). These results indicate that water stress has a certain varied effect on nodulation patterns between the two genotypes, which might contribute to the differential drought-tolerant levels of W82 and DT2008 in addition to the different root growth rate (Table 1). Moreover, one would also expect that the DT2008 would have additionally certain internal adaptation mechanisms that might enhance the symbiotic efficiency under stressful conditions.

### 3.3. DT2008 Cultivar Has a Higher Reactivation Capability Than the Model W82 Cultivar under Recovery Conditions

In the field, plants often encounter unexpected cycles of progressive soil dryness. Under such conditions, plant survival and productivity rely very much on the internal acclimatization mechanisms, which reduce or even prevent cellular damage during the stress period, as well as on the potential capacity of the stressed plant to recover and maintain normal metabolic functioning [29]. Thus, plant recovery following rewatering is an essential trait for plant survival and reflects the balance between reconstruction of damaged structures and adequate metabolism restoration [23]. Obviously, much effort has been directed towards the response of N_2_ fixation under drought conditions, while few investigations, if any, have considered the genotypic difference in nodulation and plant growth and development, particularly after recovery from progressive soil drying. Such investigation would be particularly useful for the analysis of the early changes occurring during the reactivation of normal nodule metabolic processes.

In the present report, the differential responses in the recovery from drought treatments were evidenced when DT2008 and W82 plants were subjected to 7 D of water withholding, followed by subsequent rewatering for 3 D. With the exception of nodule number per plant (Figure 3), plant rewatering was able to reduce or maintain the negative impact of drought on all nodulation and growth traits examined in the DT2008 genotype. Although in DT2008 the total nodule number per plant was not recovered in response to rewatering (Figure 3), the specific fixation per unit nodule mass would still have a chance to increase and compensate the observed reduction in nodulation number. Alternatively, the recovery time of 3 days (7 D + 3 W) was not sufficient for DT2008 to assume an effective recovery of nodule growth and development. Indeed, many small nodules were observed in DT2008 after 3 days of recovery. However, these nodules were too small in size to be considered in the data analysis. It should still be noticed that the total nodule number, as well as the total nodule FW, of DT2008 was still slightly higher than that of W82 following the recovery treatment (Figure 3 and Table 1). As for comparison of plant growth and development under recovery conditions, we found that rewatering drastically affected all of the parameters examined in W82 and DT2008. Although DT2008 and W82 were shown to be similarly affected at similar significant level upon being exposed to the recovery treatments, the DT2008 genotype remarkably exhibited a better performance when compared with W82 at the same equivalent treatment (Table 1).

## 4. Conclusions

This study aimed to characterize the newly developed soybean cultivar DT2008 when fully grown under symbiotic N_2_ fixation conditions with principle objective to determine the divergent drought responses versus the reference cultivar W82 when both are subjected to drought and recovery treatments. Contrasting tolerant and sensitive symbiotic responses were identified for each genotype in association with the microsymbiont* B. japonicum *strain USDA110. The results reported here indicated that DT2008 has a superior nodule development under water deficit and recovery in comparison with W82, highlighting that it might be a heritable trait. In addition to difference in root growth rate (this work and [14]), difference in nodule development rate might contribute to differential drought-tolerant levels of DT2008 and W82 under symbiotic conditions. Thus, DT2008 and W82 genotypes can offer a genetic resource for comparative genomics, ultimately enabling soybean scientists to identify novel SNPs and genes underlining N_2_ fixation under drought for development of soybean cultivars with improved drought tolerance. Strategies involving various omic approaches, such as transcriptomics, proteomics, and metabolomics, will be highly promising in this platform for determination of genes, mutations, and SNPs responsible for enhanced drought tolerance of DT2008 for genetic engineering. In fact, a combination of the conventional breeding, marker-assisted breeding, and genetic engineering strategies will be necessary in soybean improvement under increasing water limitation in the near future. As such, the generation of novel improved soybean cultivars bearing drought-tolerant trait(s) is highly expected to cope with the current and future expected water limitations.

## Figures and Tables

**Table 1:** Comparison of biomass production of the nodulated W82 and DT2008 plants after growth for 21 days in vermiculite soil and exposure to drought stress and recovery treatments. Well-watered (WW) plants were irrigated every day, whereas drought stress (WS) was imposed by withholding water for either four (4 D) or seven (7 D) days. Recovery treatment was experienced by withholding water for seven days followed by a subsequent rewatering for three days (7 D + 3 W). Data presented are the means ± SE of four replicates. Asterisks indicate significant differences as determined by Student's *t*-test (^*^ *P* < 0.05; ^**^ *P* < 0.01; ^***^ *P* < 0.001).

	Treatment	W82		DT2008	
		WW	WS	WW	WS
Fresh weight (g)					
Shoot	4 D	5.46 ± 0.42	3.72 ± 0.18^**^	6.92 ± 0.48	6.06 ± 0.53
	7 D	6.64 ± 0.54	3.89 ± 0.18^**^	9.94 ± 0.50	6.51 ± 0.39^**^
	7 D + 3 W	10.57 ± 0.61	7.10 ± 0.87^*^	11.03 ± 0.58	7.31 ± 0.30^**^
Root	4 D	2.32 ± 0.28	0.91 ± 0.09^**^	2.58 ± 0.19	1.78 ± 0.09^*^
	7 D	3.68 ± 0.13	1.64 ± 0.15^***^	4.11 ± 0.30	2.44 ± 0.33^**^
	7 D + 3 W	3.89 ± 0.22	2.31 ± 0.26^**^	5.62 ± 0.33	4.23 ± 0.10^**^
Nodules	4 D	0.44 ± 0.04	0.31 ± 0.03^*^	0.61 ± 0.02	0.36 ± 0.04^**^
	7 D	0.57 ± 0.05	0.27 ± 0.03^**^	0.71 ± 0.07	0.31 ± 0.03^**^
	7 D + 3 W	0.68 ± 0.06	0.35 ± 0.06^**^	0.55 ± 0.04	0.37 ± 0.02^**^

Dry weight (g)					
Shoot	4 D	0.74 ± 0.04	0.60 ± 0.03^*^	1.16 ± 0.06	1.11 ± 0.09
	7 D	1.36 ± 0.10	0.77 ± 0.04^**^	1.99 ± 0.10	1.30 ± 0.08^**^
	7 D + 3 W	2.11 ± 0.12	1.41 ± 0.18^*^	2.21 ± 0.12	1.46 ± 0.06^**^
Root	4 D	0.15 ± 0.02	0.06 ± 0.01^**^	0.18 ± 0.02	0.12 ± 0.01^*^
	7 D	0.17 ± 0.01	0.07 ± 0.01^***^	0.21 ± 0.02	0.12 ± 0.02^**^
	7 D + 3 W	0.23 ± 0.01	0.14 ± 0.02^**^	0.34 ± 0.02	0.25 ± 0.01^**^
